# An Outbreak of *Serratia marcescens* in a Cardiothoracic Surgery Unit Associated with an Infected Solution of Pre-Prepared Syringes

**DOI:** 10.3390/antibiotics14030319

**Published:** 2025-03-18

**Authors:** Maria Papagianni, Eleni Mylona, Sofia Kostourou, Chrysoula Kolokotroni, Dimitris Kounatidis, Natalia G. Vallianou, Vasileios Papastamopoulos

**Affiliations:** 1 Fifth Department of Internal Medicine and Infectious Diseases Unit, Evangelismos General Hospital, 10676 Athens, Greece; marianthi.p@gmail.com (M.P.); mylonaelena@gmail.com (E.M.); vpapastamopoulos@evaggelismos-hosp.gr (V.P.); 2 Infection Prevention Unit, Evangelismos General Hospital, 10676 Athens, Greece; skostourou@gmail.com (S.K.); ckolokotroni@gmail.com (C.K.); 3 Diabetes Center, Medical School, National and Kapodistrian University of Athens, Laiko General Hospital, 11527 Athens, Greece; dimitriskounatidis82@outlook.com; 4 First Department of Internal Medicine, Sismanogleio General Hospital, 15126 Athens, Greece

**Keywords:** *Serratia marcescens*, bacteremia, outbreak, syringes, infection and prevention control measures

## Abstract

**Background/Objectives:** The aim of the present study is to report an outbreak of bloodstream infections caused by *Serratia marcescens* in patients undergoing postoperational procedures in the Cardiothoracic Department and to describe the epidemiological investigations and control measures undertaken. A cluster of bacteremia due to *Serratia marcescens* was identified in blood cultures from postoperative patients in the Cardiothoracic Surgery Department in November 2023. **Methods:** Active surveillance by the hospital’s prevention and control team was initiated. Interviews with nurses and sanitary personnel and reviews of the most common procedures, such as hand washing, bladder catheterization, and intravenous catheter care, were performed. Culturing samples from hospital personnel, postoperative patients, and the environment, including pressure transducers, tap water, soap, therapeutic solutions, antiseptics, respirators, and various intravenous preparations, were drawn up. Overall, 225 samples were collected, including 149 blood cultures, and these were all sent to the Hospital’s Microbiology Laboratory. **Results:** Twenty-three out of forty-seven postoperative patients had positive blood cultures for *Serratia marcescens.* All the postoperative patients involved in the outbreak received cefepime according to antimicrobial susceptibility testing. Three pre-prepared flushing syringes were found to be positive for *Serratia marcescens* as well. The Cardiothoracic Department was kept under surveillance with hand hygiene measures, infusion preparation, medical device use, and cleaning procedures reviewed by the infection’s prevention and control team. **Conclusions:** Undoubtedly, nosocomial outbreaks represent an important health issue regarding morbidity, mortality, and costs. Timely interventions by the hospital’s infection prevention and control team may be life-saving under these circumstances.

## 1. Introduction

*Serratia marcescens (S. marcescens)* is a ubiquitous Gram-negative bacterium, which belongs to the *Enterobacterales* order [1,2]. *S. marcescens* is a motile, facultative anaerobe rod, that due to endogenous factors, possesses the ability to adapt to different environments, such as the air, water, plants, and hospital settings [3,4]. As it may easily adapt to the context of a hospital, it is considered an opportunistic pathogen causing infections to immunocompromised patients, especially to neonates [3,4,5]. The first outbreak of *S. marcescens* dates back to 1950 in San Francisco, where 11 patients were diagnosed with urinary tract infections due to *S. marcescens* [6]. According to the Outbreak Database, the largest collection of nosocomial outbreaks available, there were 103 reports of *S. marcescens* outbreaks between 1968 and 2010 [7]. *S. marcescens* has been alleged to be the fifth most common cause of nosocomial outbreaks worldwide [7] More recently, the European Center for Disease Prevention and Control (ECDC) has reported *S. marcescens* to be the ninth most frequently isolated cause of bloodstream infections (BSIs) in the ICU in the European Union [8]. Hospital outbreaks have been linked to contaminated intravenous fluids, compounded drugs, heparin vials, hospital soaps, and disinfectants as well as the consumption of tap water [9,10,11,12]. In addition, the lack of preventive infection control measures seems to be of paramount importance. In this context, the hands of the healthcare workers seem to be a common vehicle for further transmission and spread of *S. marcescens.*

The spectrum of infections caused by *S. marcescens* includes, but is not limited to, bacteremia, endocarditis, pneumonia, osteomyelitis, arthritis, meningitis, and endophthalmitis [13,14,15,16,17]. This pathogen is more frequently isolated from the lower respiratory tract as a cause of pneumonia and from blood cultures as a cause of sepsis [17]. Treatment of *S. marcescens* infections is frequently demanding, mainly because the pathogen exhibits intrinsic antibiotic resistance and often expresses antimicrobial multidrug-resistant (MDR) phenotypes [18].

The purpose of this study was to describe the outbreak of *S. marcescens* in a 5D Cardiothoracic Surgery Department, which was contained in a timely manner due to the cooperation of the Infectious Diseases Department, the Microbiology Laboratory, and the Infections Control and Prevention Nurses (ICP) Team of our hospital.

## 2. Results

On 13th November 2023, the chief nurse of the 5D Cardiothoracic Surgery Department reported to the Infectious Diseases Department the unusual event of five patients with fever and rigor. The patients had developed a fever over the previous few days, and blood cultures had already been conducted. Later, during the same day, the Microbiology Laboratory also reported to the Infectious Diseases Department that six blood cultures from the 5D Cardiothoracic Surgery Department were positive for *S. marcescens*.

### 2.1. Epidemiological Investigation

Forty-seven patients were registered in the department on the day of the first notification of the outbreak. According to the registered patient movement, the outbreak was restricted to the 5D Cardiothoracic Surgery Department, which is in the Cardiothoracic Ward, and did not include the Cardiothoracic ICU or other departments of the hospital. Registration of invasive devices and catheters was conducted with assistance by an audit tool. According to the registration, all the catheters of the postoperative patients were “closed”, which means that there was no continuous intravenous infusion, and catheter patency was secured with periodical heparin flushing.

Infectious disease counseling was conducted for all the postoperative patients with a positive blood culture, and instructions for antibiotic treatment and its duration were given depending on whether a patient had a foreign body or not (mechanical heart valve, cardiac implantable device, and/or other). All postoperative patients with a positive blood culture received cefepime according to the susceptibility testing results from the positive blood cultures, as reported by our hospital’s Microbiology Laboratory.

New blood cultures were sent on the following three days for all the postoperative patients. For postoperative patients with a previously positive blood culture, the new blood culture was used for the follow up and the response to treatment. For the postoperative patients that previously had a negative blood culture, the new blood culture was used for surveillance. There was one patient with a positive surveillance culture but with no fever. Concern regarding other asymptomatic patients, who could develop fulminant bacteremia, was raised. Nevertheless, this outbreak was successfully contained.

Altogether, 23 postoperative patients with *S. marcescens* bacteremia were detected among the 47 patients in the 5D Cardiothoracic Surgery Department. Nineteen postoperative patients completed 7 days of treatment and were discharged, and three patients completed their treatment for the bacteremia but remained hospitalized due to other reasons. Only one patient was intubated and transferred to the ICU of another hospital nearby.

To identify possible reservoirs, the place of preparation for the intravenous infusions was inspected and investigated. There was a sink for hand hygiene close by; however, the trolleys and the preparation of the infusions were kept at a safe distance from the sink, i.e., 2 to 3 m away. Moreover, trolleys for nursing and surgical trauma changing were inspected, and it was confirmed that they were all overloaded and they all had a glass container full of ready-to-use 2.5 mL syringes for the flushing of the patients’ intravenous catheters.

The Cardiothoracic Department was subjected to more intensive surveillance and instructions. Due to the simultaneous onset of several cases of *S. marcescens* bacteremia, a source related to intravenous drug or fluid distribution was suspected, and thus, surveillance of device use and care for intravenous catheters were a particular focus for the ICP Nurse Team.

### 2.2. Microbiological Investigation

To identify possible reservoirs, extensive environmental sampling was undertaken, and cultures were received from preparation material and equipment for the intravenous (iv) fluids: salines for infusion, devices for iv infusions, syringes ready for iv catheter flushing, multi-dose heparin flacon, ready-to-use drug infusions, and liquid soaps for hand hygiene. The multi-dose heparin vial contained 25,000 Units/5 mL, and if we consider that 2.5 mL syringes were used with 1 mL of fluid drawn, we can imagine that these vials could have been used five times.

After 48 h from the first environmental sampling, the Hospital’s Microbiology Laboratory reported that three prepared and ready-to-use heparin syringes for intravenous catheter flushing tested positive for *S. marcescens*.

### 2.3. General Management of the Bacteremia Due to the S. marcescens Outbreak

All 23 postoperative patients with *S. marcescens* bacteremia had undergone heparin flushing procedures in which filled syringes were used. It was presumed that the source of the outbreak was a multi-dose heparin vial that was contaminated during the preparation of the syringes used for catheter iv flushing. Despite laborious effort, the multi-dose flacon source was ultimately not found. Whether the initial source was an index patient or an environmental niche of the microorganism remained elusive.

A team from the National Organization for Public Health visited our hospital in order to inspect the department of the outbreak and participated in a meeting aiming to coordinate all persons involved in the management of the outbreak. Joined forces from the National Organization of Public Health and the ICP Nurse Team from our hospital collected samples for environmental surveillance and colonization samples. In total, 50 environmental samples were collected. Five samples were collected from the hands of healthcare workers from the outbreak department. *S. marcescens* was not detected in any of the samples, neither from the environment nor from healthcare personnel’s hands, while no other pathogens were isolated.

The frequency of cleaning was augmented for the 5D Cardiothoracic Surgery Department. All the wards were decontaminated with chlorine water at a concentration of 2.4 g in a dilution of 2 tabs in 2.4 L of water. Following manual decontamination, the next step was decontamination by nebulization with hydrogen peroxide.

During the following weeks, there were no other cases of *S. marcencens* bacteremia or other site infections from this or any other outbreak pathogen.

Table 1 and Table 2 depict the main characteristics of the *S. marcescens* outbreak in the Cardiothoracic Surgery Department.

## 3. Discussion

Our study reports an overwhelming outbreak of 23 bacteremia cases associated with prepared heparin flushing solutions, contaminated by *S. marcescens*. *S. marcescens,* which is known for its opportunistic nature, causes invasive infections mainly to immunocompromised or frail hosts in a hospital setting. The variables associated with the spread of HAIs comprise inadequate infection control practices alongside the expanding array of contemporary medical procedures. Environmental and personnel sampling paired with the epidemiological investigation pointed towards prefilled heparin catheter flushes, as the cause of this *S. marcescens* bacteremia outbreak. In spite of this meticulous investigation, the actual initial source of the outbreak remained unclear. The general measures of environmental cleaning, reinforcing iv preparation hygiene, and enhanced surveillance of the department ceased the outbreak, and no further cases were reported.

It is noteworthy that the head nurse perceived and reported the unusual event of several postoperative patients having fever simultaneously and referred them immediately to the Hospital’s Infectious Diseases Department. Concurrently, the Microbiology Laboratory alerted the Infectious Diseases Department about the outbreak on the same day of *S. marcescens* isolation. The outbreak investigation commenced immediately upon notification. A comprehensive literature review and a thorough conversation between physicians, nurses, and the hospital infection control nurses were undertaken promptly. The team reflected on all plausible ecological niches for *S. marcescens* in the department, before the actual environmental sampling.

Central venous catheters (CVCs) are utilized for extended durations and require regular flushing with saline and heparin to maintain their functionality. This prolonged use and frequent flushing increase the likelihood of patients developing BSIs due to contaminated flushing solutions [18,19,20]. However, this outbreak illustrates that the timeframe between exposure to a contaminated flushing solution and the onset of a BSI may vary between patients.

The contamination could have been introduced erroneously throughout the distinct stages of the preparation technique. The usage of multiple doses of heparin vials may have contributed to the spread of *S. marcescens* among patients in this Cardiothoracic Department.

The epidemiologic study supported this conclusion, despite the lack of microbiologic evidence of contamination from environmental and product testing. Consequently, after the investigation, continuous monitoring and education in the Cardiothoracic Department were undertaken. No other similar infection cases were reported until this time.

In several studies regarding nosocomial outbreaks, a single-strain reservoir was epidemiologically suspected but rarely confirmed. There is a consensus that the primary focus of outbreak investigations should be on resolving the issue to eliminate any further patient risk. Nevertheless, despite the thorough investigation and the recognition of the distribution pathway of the outbreak, we could not identify the exact niche of the microorganism. Nevertheless, early investigation and distribution pathway recognition led to the prevention of further cases. In order to prevent a potential future outbreak, infection control measures with the reinforcement of intravenous solution preparations and hygiene precautions are of the utmost importance. As a matter of fact, the exact pathways of transmission in nosocomial outbreaks have scarcely been recognized and demonstrated comprehensibly. Our study, in line with other similar studies, demonstrates the role of environmental sources of *S. marcescens* epidemics and the perils of lapses in the aseptic technique during the use of medication vials on multiple patients [18,19,20].

Patients such as post-cardiothoracic surgery patients are especially susceptible to infections due to various parameters. Prolonged transmission opportunities as a result of the complexity of modern cardiothoracic procedures, together with prior hospitalizations and the administration of antimicrobial agents, are the most well-recognized factors [18,19,20,21,22]. In addition, other risk factors, namely diabetes mellitus, chronic obstructive pulmonary disease, obesity and re-operation due to bleeding, the length of ICU stay, and tube feeding, have also been associated with *S. marcescens* infection or colonization [20,21,22,23,24].

In any nosocomial outbreak, it is crucial to detect the origin of the infection and concurrently to unveil the roots of transmission. Having secured the above, it is important to define the most relevant measures necessary to halt the outbreak. *S. marcescens* outbreaks are characterized by diverse origins and complex mechanisms of transmission, and thus, occasionally, the outbreak origin remains unclear [21,22,23,24,25,26,27,28,29,30].

There are only three reports of *S. marcescens* outbreaks in Cardiothoracic Surgery Department Units, and these were reported prior to 2010. However, nowadays, there are various reports of outbreaks of *S. marcescens* in the hospital setting, especially in neonatal ICUs [31,32,33]. Factors associated with *S. marcescens* acquisition or infection among neonates include low birth weight, CVCs, and prior administration of broad-spectrum antibiotics, together with the existence of concomitant underlying diseases [5,31,32,33]. Fewer studies have been conducted in other departments apart from ICUs, despite the fact that surgical departments and postoperative patients in particular are also vulnerable to nosocomial outbreaks. Notably, outbreaks due to *S. marcescens* may be particularly difficult to control due to multiple reasons, such as the easy adaptation of this bacterium to various environments, such as hospital wards, together with the MDR nature that *S. marcescens* exhibits.

We would like to underscore that the rapid termination of the outbreak was accomplished through direct collaboration between the hospital ICP Nurse Team, the Cardiothoracic Surgery Department, and the Microbiology Laboratory. The findings of the investigation led to a reinforcement of hygiene training, correction of problematic practices, and high vigilance for disinfection procedures in order to ensure the end of the current outbreak and the avoidance of similar outbreaks in the near future.

Initially, in an outbreak of *S. marcescens* or other microorganisms, several source types need to be considered: a single local source, e.g., a soap dispenser from which the bacterium is spread to other patients, a patient infected or colonized with *S. marcescens*, or contaminated iv fluids or drugs [29,30,31,32,33,34,35,36]. In our study, due to the simultaneous cases of bacteremia, contamination related to infusions administered intravenously was suspected in a timely manner.

As aforementioned, *S. marcescens* outbreaks have long been documented in the medical literature. According to the source of the outbreak, these outbreaks have been categorized as single-source, multiple-source, and unknown-source outbreaks. Soap dispensers, contaminated chlorhexidine, contaminated brushes and razors and preoperative shaving, haircutting toolkits, ultrasonography and echocardiography probes, and bronchoscopes are well-documented sources of outbreaks [29,30,31,32,33,34,35,36]. Moreover, several *S. marcescens* outbreaks have been attributed to iv drugs and infusions, including, but not limited to, propofol vials, insulin, furosemide solutions, and opioid solutions [37,38,39,40,41,42,43,44,45,46]. Outbreaks due to patients with a high colonization index and infection control breaks in the unit have also been reported [47].

Multiple additional studies document instances of contaminated heparin vials leading to outbreaks [48,49,50,51]. Intrinsic contamination of heparin has been reported in the past, involving products contaminated during pharmaceutical compounding rather than during the manufacturing process [48,49]. Identifying pharmaceutical contamination can be difficult due to its often irregular occurrence within a specific batch. In our study, we could not identify a contaminated vial; thus, our hypothesis leaned towards contamination during heparin flushing preparation. However, outbreaks of *S. marcescens* bacteremia due to contaminated prefilled heparin or saline syringes have also been documented in the medical literature.

Genome analysis has revealed that strains related to healthcare facilities have been isolated worldwide and manifest in a vast repertoire of antimicrobial resistance mechanisms [52,53,54,55,56,57]. Regarding outbreaks of *S. marcescens*, this bacterium has intrinsic resistance to a variety of antimicrobial agents, such as amoxicillin, ampicillin, amoxicillin–clavulanate, ampicillin–sulbactam, first- and second-generation cephalosporins, polymyxins, nitrofurantoin, and macrolides. Moreover, lately, *S. marcescens* has exhibited acquired resistance even to third- and fourth-generation cephalosporins and carbapenems. Carbapenem-resistant *S. marcescens* is mainly attributed to class A, especially KPC, class B, particularly NDM metalloenzymes, and class D carbapenemases, mainly OXA-48 [52,53,54,55,56,57]. Therefore, treatment options are frequently narrowing on account of AMR. It is noteworthy that in our study on this Cardiothoracic Department *S. marcescens* outbreak, *S. marcescens* was susceptible to cefepime, which was also documented to be efficacious in the clinical setting. Thus, hygiene techniques, together with the lack of resistance to cefepime, proved to be beneficial in containing this outbreak as well. Notably, due to the aforementioned factors, BSI outbreaks due to *S. marcescens* have recently been accompanied by a high mortality rate. In particular, it has been reported that in 2022 in Hungary, among eight patients in an ICU with documented BSIs due to an *S. marcescens* outbreak, five died. This increased mortality rate, i.e., 62.5%, occurred despite the fact that the two circulating Pulsed-Field Gel Electrophoresis (PEGF) types of *S. marcescens* isolated in this BSI outbreak did not exhibit MDR. However, these two PFGE types of *S. marcescens* were resistant to the disinfectant that is usually used in this ICU, i.e., quaternary ammonium. When the quaternary ammonium was substituted by another disinfectant and the hospital undertook several other prevention control measures, mainly a strict protocol regarding hand hygiene, they managed to contain the outbreak, with no further cases reported [52]. In other studies, mortality regarding BSIs caused by *S. marcescens* has been reported to range between 14% and as high as 85% [52,53,54,55,56,57]. Therefore, this ubiquitous bacterium may be particularly difficult to combat, as it may survive in the hospital environment when commonly applied disinfectants are utilized. This adaptability of *S. marcescens* is highly attributed to its genetic predisposition to be resistant to various disinfectants, in addition to its AMR to a variety of antimicrobial agents.

ICPs typically utilize the results of positive bacterial blood cultures as a screening method to identify potential HAI cases. Nevertheless, conducting surveillance manually is not only labor-intensive but also prone to certain HAIs being overlooked. Nowadays, automated detection systems have been developed to integrate information from various sources, including microbiology and other laboratory findings, as well as medical and medication records [58]. Unfortunately, our medical facility lacks a system for real-time monitoring of infections. As the current prevalence of HAIs in Greece remains an important issue, such systems in routine practice would be highly beneficial and should be further encouraged. In addition, despite the remarkable work of the ICP Nurse Team in our hospital, there is still a scarcity of infection prevention practitioners, and there is no special department with allocated resources. This is a healthcare issue that needs to be further investigated and should not be underestimated.

Furthermore, the role of the Microbiology Laboratory in the early detection of the emergence or clusters of unusual microorganisms and resistance patterns should be emphasized. The continuous monitoring and data analysis of routine cultures ensure a prompt alert of the occurrence of unusual events. Close collaboration and communication between ICPs and the laboratory personnel can therefore help to detect and eliminate potential outbreaks of HAIs. The present study is another example, demonstrating how the microbiology personnel of our hospital assisted in infection control. The outstanding professional awareness of the Clinical Microbiology Laboratory personnel, together with the remarkable communication between the laboratory and the ICPs in alliance, promoted the early detection of the outbreak, helped in conducting an efficient investigation, and led to the prompt termination of the outbreak.

Nowadays, there are computer-assisted surveillance systems, automatic or semi-automatic, which utilize microbiological data, registration data, and pharmacy data, which are integrated in order to detect potential outbreaks of HAIs. Consequently, the integration of such information technology would enhance infectious disease surveillance. Notably, high-frequency computer-assisted point prevalence surveys could play a crucial role in hospital infection control and outbreak detection. This technology could help detect even subtle changes in the point prevalence of HAIs in a timely manner [58].

## 4. Patients and Methods

### 4.1. Hospital Setting

The General Hospital of Evangelismos is a tertiary 950-bed hospital. The outbreak occurred in the 5D Cardiothoracic Surgery Department and did not affect the specific Cardiothoracic ICU, nor was it initiated in the Cardiothoracic ICU. Patients admitted to the Cardiothoracic ICU of our hospital are pre- or post-cardiothoracic surgery subjects who need close monitoring. In the 5D Cardiothoracic Surgery Department, there are postoperative patients who should be monitored after their operation and/or after their stay in the Cardiothoracic ICU. Notably, the daily number of nurses per patient in the wards varies between 0.25 and 1.

Active hospital-wide surveillance for Hospital-Acquired Infections (HAIs) was conducted by the Infection Control Team at Evangelismos hospital. The Infection Control Team consists of Infection Control and Prevention (ICP) nurses that conduct point prevalence studies and practice surveillance for MDR bacteria. The ICP Team collaborates and is part of the Infectious Diseases Department, which is a cross-functional department with infectious disease counseling and ICP duties. This study was conducted according to the Declaration of Helsinki for human beings. In addition, it received approval from the Ethical Committee of Evangelismos General Hospital, with the protocol number 91/28-02-2025.

### 4.2. Sample Collection

Extensive environmental sampling was performed. Screening samples were taken with swabs. Environmental samples were taken from the 5D Cardiothoracic Surgery Department used for the postoperative care of cardiac surgery patients. Blood cultures were drawn from all the postoperative patients, irrespectively of fever, in the 5D Cardiothoracic Surgery Department and this Cardiothoracic ICU. More specifically, 225 samples were sent to the Hospital’s Microbiology Laboratory, amongst which 149 were blood cultures. Fifty samples were taken from pressure transducers, tap water, soap, therapeutic solutions, antiseptics, respirators, and various other intravenous preparations. In addition, 5 samples were collected from the hands of healthcare workers in the 5D Cardiothoracic Surgery Unit.

### 4.3. Epidemiological Investigation

Bloodstream infection (BSI) is defined as laboratory-confirmed with positive blood culture infection in a patient with signs and/or symptoms of systemic infection. In our study, a case was defined as a postoperative patient with *S. marcescens* BSI with a positive blood culture that was obtained more than 48 h after being admitted to the hospital.

The usual flow of the patients was between the Operation Theater, the Cardiothoracic ICU, and the 5D Cardiothoracic Surgery Department. Thus, it was decided to cohort and pause for all new admissions in the 5D Cardiothoracic Surgery Department. However, there was no disturbance in the flow of cardiothoracic surgery as the pre-surgery and post-surgery hospitalization of the newly admitted patients was appointed to a neighboring department of the hospital.

In order to localize the outbreak, investigate its origin, and understand its spread in the hospital, we registered all postoperative patients’ movements during the 5 days prior, irrespectively of whether they had a fever or not. Moreover, the registration of temperature for all the postoperative patients in the department for 5 days before the acknowledgment of the problem and the registration of devices and catheters for all the postoperative patients in the department using audit tools were also undertaken.

An upper respiratory specimen for Rapid-Ag COVID-19 was collected from all the postoperative patients in the department irrespective of whether they had a fever. Furthermore, the preparation of the intravenous infusion and trolleys for nursing was inspected. Moreover, extensive discussions were held with the chief nurses and doctors of the department; however, there were no structured interviews.

### 4.4. Microbiological Investigation and Antimicrobial Susceptibility Testing

A total of 225 samples were sent to the Hospital’s Microbiology Laboratory. Blood cultures were further analyzed with incubation for up to 5 days in the BacT/ALERT 3D culture system (Becton Dickinson, Sparks, MD, USA). A subculture of positive blood cultures was performed on blood agar, chocolate agar, and MacConkey agar. Environmental samples were also cultured on blood, chocolate, and MacConkey agar. *S. marcescens* grew on the culture media after 24 h of incubation aerobically at 36 °C in a 5% CO_2_-enriched atmosphere. Identification was performed using matrix-assisted laser desorption ionization–time-of-flight mass spectrometry (MALDI-TOF MS) with the Vitek MS system (bioMerieux, Craponne, France). Antimicrobial susceptibility testing was performed by using the automatic Vitek 2 Compact System (bioMerieux, Craponne, France). Interpretation of the results was performed according to the latest guidelines of the European Committee on Antimicrobial Susceptibility Testing (EUCAST). Isolates were tested on the following antibiotics: ampicillin, amoxicillin/clavulanic acid, ampicillin/sulbactam, ticarcillin, piperacillin, piperacillin/tazobactam, cefotaxime, ceftazidime, ceftriaxone, cefepime, aztreonam, ertapenem, meropenem, amikacin, gentamicin, tobramycin, ciprofloxacin, levofloxacin, moxifloxacin, minocycline, tetracycline, colistin, and trimethoprim/sulfamethoxazole. Among the antibiotics evaluated, *S. marcescens* showed intrinsic resistance to ampicillin, amoxicillin/clavulanic acid, ampicillin/sulbactam, and colistin. Moreover, all examined isolates were susceptible to cefepime (MIC ≤ 1 mg/L), which was the therapeutic choice for all the patients with positive blood cultures.

## 5. Conclusions

In conclusion, we have reported on an unusual outbreak of *S. marcescens* BSIs in a Cardiothoracic Surgery Department. The contamination of a prefilled heparin syringe was associated with the outbreak. The intersectional collaboration within the hospital led to a prompt investigation and, ultimately, the relatively fast termination of the outbreak. The reinforcement of infection control policies, the continuous personnel education on aseptic drug preparation procedures, and the vigilance of the ICP Nurse Team together with laboratory personnel cooperation are important measures for the timely termination of an outbreak. Continuous education on infection control policies and monitoring of aseptic preparation procedures are necessary for all healthcare personnel responsible for the rapid identification and containment of such outbreaks. In this context, the implementation of computer-assisted technology should not be overlooked, as it may lead to the detection an outbreak in its early stages.

## Figures and Tables

**Table 1:** The main features of this outbreak.

Summary of the Outbreak	
Where	5D Cardiothoracic Surgery Department
Duration of the outbreak	9 days
Day 1	10 November 2023
Day 9	18 November 2023
Total number (Nb) of patients on day 1	47
Nb of patients with *S. marcescens* bacteremia	23
Nb of patients with *S. marcescens* bacteremia that received antibiotic treatment	23
Patient with negative blood culture on day 2	17
Patients with *S. marcescens* bacteremia and body temperature > 37.5 °C	3
Patients with *S. marcescens* bacteremia without preceding surgical intervention	3
Total number of blood cultures	149

**Table 2:** The new cases that occurred and the days on which these new cases of the outbreak took place.

Days	1	2	3	4	5	6	7	8	9
New cases of *S. marcescens* bacteremia	**2**	**0**	**5**	**13**	**0**	**1**	**0**	**0**	**2**

Notably, day 3 was the first day the ICP Team was alerted.

## Data Availability

The original contributions presented in this study are included in the article. Further inquiries can be directed to the corresponding authors.
